# Record-Breaking Early Flowering in the Eastern United States

**DOI:** 10.1371/journal.pone.0053788

**Published:** 2013-01-16

**Authors:** Elizabeth R. Ellwood, Stanley A. Temple, Richard B. Primack, Nina L. Bradley, Charles C. Davis

**Affiliations:** 1 Department of Biology, Boston University, Boston, Massachusetts, United States of America; 2 Department of Forest and Wildlife Ecology, University of Wisconsin, Madison, Wisconsin, United States of America; 3 Aldo Leopold Foundation, Baraboo, Wisconsin, United States of America; 4 Department of Organismic and Evolutionary Biology, Harvard University Herbaria, Cambridge, Massachusetts, United States of America; Cirad, France

## Abstract

Flowering times are well-documented indicators of the ecological effects of climate change and are linked to numerous ecosystem processes and trophic interactions. Dozens of studies have shown that flowering times for many spring-flowering plants have become earlier as a result of recent climate change, but it is uncertain if flowering times will continue to advance as temperatures rise. Here, we used long-term flowering records initiated by Henry David Thoreau in 1852 and Aldo Leopold in 1935 to investigate this question. Our analyses demonstrate that record-breaking spring temperatures in 2010 and 2012 in Massachusetts, USA, and 2012 in Wisconsin, USA, resulted in the earliest flowering times in recorded history for dozens of spring-flowering plants of the eastern United States. These dramatic advances in spring flowering were successfully predicted by historical relationships between flowering and spring temperature spanning up to 161 years of ecological change. These results demonstrate that numerous temperate plant species have yet to show obvious signs of physiological constraints on phenological advancement in the face of climate change.

## Introduction

The sensitivity of flowering times to temperature has proven valuable for investigating the impacts of climate change on plants [1]–[3]. Plant phenology appears to have largely kept pace with warmer temperatures, with numerous species flowering earlier now than in the past. However, recent years have seen record-breaking spring temperatures that are well outside the realm of historical trends [4], [5]. Although flowering dates for many responsive species have greatly advanced with warmer temperatures, at some point plants may no longer flower earlier in response to warming due to photoperiod constraints or unmet winter chilling requirements [6]–[8]. Extreme weather events such as those observed in the eastern United States in 2010 and 2012 provide opportunities to determine if historical phenological responses to rising temperatures are maintained under novel conditions presented by very recent climate change.

Changes in plant phenology have broad implications at the ecosystem level. Flowering and leafing out times signal the start of the growing season, and altered phenology influences associated ecosystem processes such as nutrient cycling and carbon sequestration [9], [10]. Interactions with herbivores, pollinators, and other ecological associates may be compromised and lead to ecological mismatches [11]–[15]. Also, advanced spring phenology, followed by late frost events, can damage flowers and young leaves, which has negative impacts on plant growth and fruit development [16]–[18]. Finally, warmer temperatures can also expose plants to drought, resulting in decreased reproductive success [19].

Two of the best-known American environmental writers initiated extensive phenological observations of flowering times in the eastern United States that encompass 161 years of ecological change. From 1852–1858, Henry David Thoreau, author of *Walden* [20], observed flowering times in Concord, Massachusetts, USA. And from 1935–1945, Aldo Leopold, author of *A Sand County Almanac* [21], recorded flowering times in Dane County, Wisconsin, USA and near the site of his “Shack” in adjacent Sauk County [4]. Several recent re-surveys at these locations [22]–[25], nearly 1500km apart, indicate that many spring-flowering plants now flower much earlier than in the past. This trend appears to be attributable to especially warmer spring (March, April, May) temperatures [25]–[27]. In 2010 and 2012 in Massachusetts [5], and 2012 in Wisconsin [4], spring temperatures were the warmest on record. These long-term datasets thus provide a rare opportunity to investigate if historical relationships between flowering times and spring temperatures apply during these record-breaking years. These observational data are especially timely because recent meta-analyses of flowering phenology [28] have documented that controlled warming experiments greatly under-predict flowering phenology when compared with their responses in natural settings. Thus, historical phenological data, such as those initiated by Thoreau and Leopold, are critical to understanding plant responses to current and future warming, and to test whether increasing temperatures may result in continued earlier flowering.

## Results and Discussion

**Figure 1:**
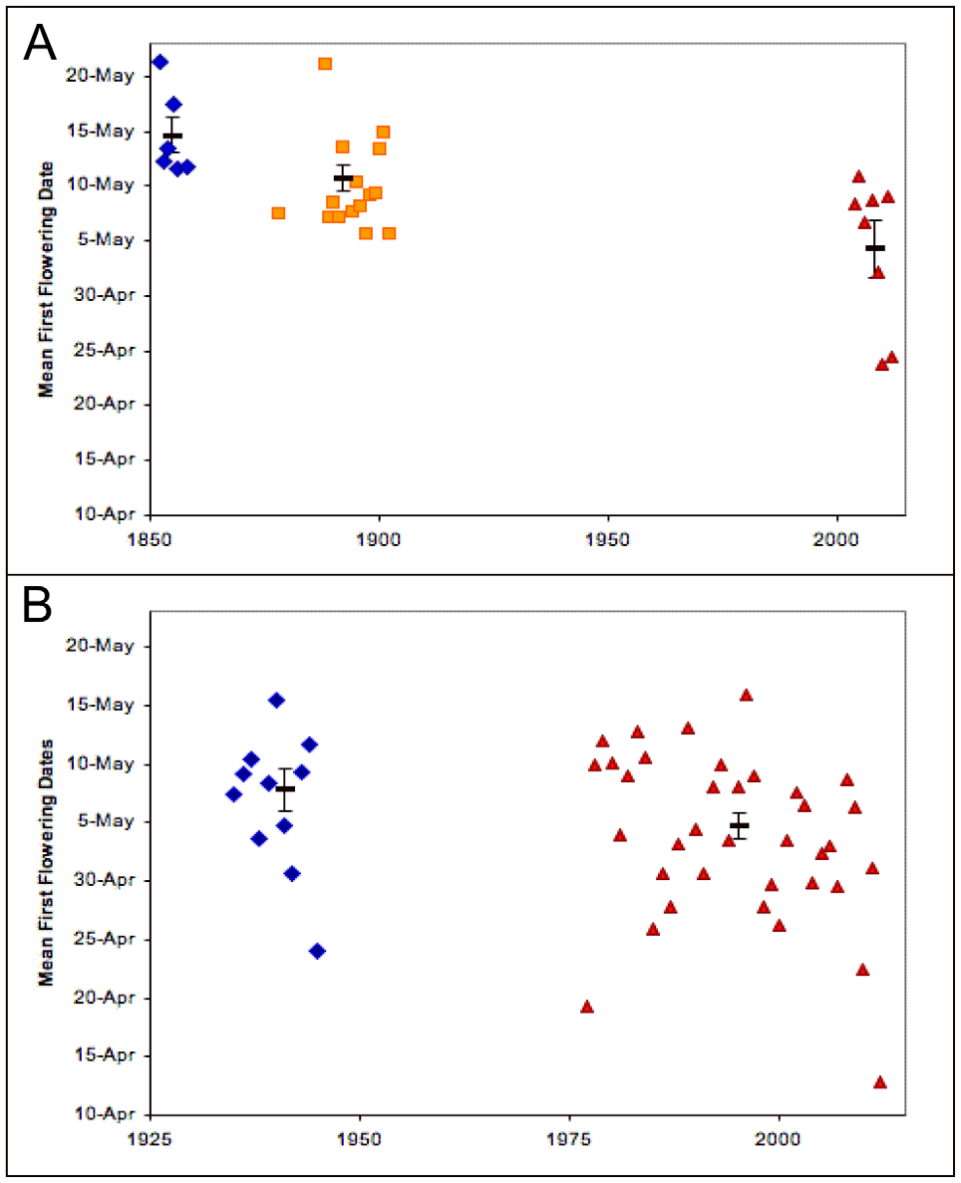
Mean first flowering dates for all species. The annual mean first flowering dates and standard errors of 29 years of data are shown from Massachusetts (a) and 47 years from Wisconsin (b). Blue triangles = Thoreau and Leopold et al.; orange squares = Hosmer; red triangles = Primack et al. and Bradley et al..

In Concord, Massachusetts, 32 spring flowering native plant species representing a broad phylogenetic diversity were chosen because they were observed in nearly all of the following 29 years: 1852–1858, 1878, 1888–1902, 2004–2006 and 2008–2012 [24] (Fig. 1a; Table 1, and phylogenetic relationships in Figures S1a and S1b). From 1852–1858, when mean spring temperature in the region was 5.5°C, mean first flowering date for these species was 15 May. By 1878–1902 their mean first flowering date had shifted five days earlier to 10 May, when mean spring temperature was 6.3°C. During the past nine years mean first flowering has shifted to 4 May, 11 days earlier than in Thoreau's time and during a period in which mean spring temperature has risen to 8.8°C. Warming in the greater Boston area, which includes Concord, has been attributed to both global warming and the urban heat island [29]. Within the past decade, two years have been record breakers in this region: mean spring temperature in 2010 was the warmest ever recorded at 11.0°C, during which time plants had a mean flowering date of 24 April; and 2012 was the second warmest spring on record at 10.7°C, during which time plants had a mean flowering date of 25 April. In these two years, plants flowered three weeks earlier (i.e., 21 and 20 days in 2010 and 2012, respectively) than when Thoreau observed them in Concord.

**Table 1:** List of plant species monitored at each location, along with their phenological responses to temperature (for years prior to 2010 for MA and prior to 2012 for WI) and 95% prediction intervals for 2010 and 2012 for Massachusetts and 2012 for Wisconsin.

Location	Species	n	Temp. Response	95% Prediction Interval	Obs. FFD
Massachusetts	*Amelanchier arborea* *	25	y = −3.24x+143	2010: 95–119	2010: 102
	(Serviceberry)		R^2^ = 0.45***	2012: 96–119	2012: 105
Massachusetts	*Anemone quinquefolia* *	25	y = −1.03x+123	2010: 96–127	2010: 102
	(Wood Anemone)		R^2^ = 0.05*	2012: 97–127	2012: 105
Massachusetts	*Aquilegia canadensis* *	25	y = 0.46x+112	2010: 97–138	2010: 105
	(Wild Columbine)		R^2^ = 0.01	2012: 98–137	2012: 109
Massachusetts	*Aralia nudicaulis*	24	y = −3.27x+160	2010:113–134	2010: 122
	(Wild Sarsaparilla)		R^2^ = 0.68***	2012: 114–135	2012: 128
Massachusetts	*Arenaria lateriflora*	23	y = −4.92x+178	2010: 105–142	2010: 127
	(Bluntleaf Sandwort)		R^2^ = 0.43***	2012: 107–143	2012: 123
Massachusetts	*Caltha palustris* *	25	y = −1.69x+116	2010: 75–120	2010: 69
	(Marsh Marigold)		R^2^ = 0.06**	2012: 76–120	2012: 92
Massachusetts	*Comandra umbellate*	24	y = −3.26x+162	2010: 107–145	2010: 124
	(Bastard Toadflax)		R^2^ = 0.24***	2012: 108–145	2012: 128
Massachusetts	*Cornus canadensis*	24	y = −3.27x+164	2010: 114–142	2010: 136
	(Dwarf Dogwood)		R^2^ = 0.36**	2012: 116–143	2012: 138
Massachusetts	*Cypripedium acaule*	25	y = −3.70x+165	2010: 110–138	2010: 124
	(Pink Lady Slipper)		R^2^ = 0.61***	2012: 112–138	2012: 123
Massachusetts	*Fragaria virginiana* *	20	y = −4.21x+152	2010: 85–127	2010: 105
	(Wild Strawberry)		R^2^ = 0.33***	2012: 87–128	2012: 105
Massachusetts	*Gaylussacia baccata*	24	y = −5.82x+174	2010: 97–123	2010: 115
	(Black Huckleberry)		R^2^ = 0.68***	2012: 99–125	2012: 109
Massachusetts	*Geranium maculatum* *	25	y = −1.85x+151	2010: 117–143	2010: 129
	(Wild Geranium)		R^2^ = 0.16**	2012: 118–144	2012: 128
Massachusetts	*Houstonia caerulea*	26	y = −2.70x+127	2010: 78–117	2010: 86
	(Bluet)		R^2^ = 0.17**	2012: 79–118	2012: 92
Massachusetts	*Hypoxis hirsuta*	25	y = −3.31x+168	2010: 117–146	2010: 129
	(Yellow Star-Grass)		R^2^ = 0.34***	2012: 119–147	2012: 128
Massachusetts	*Krigia virginica*	24	y = −4.21x+171	2010: 99–151	2010: 115
	(Dwarf Dandelion)		R^2^ = 0.22***	2012: 101–151	2012: 109
Massachusetts	*Potentilla canadensis*	24	y = 0.21x+116	2010: 89–148	2010: 102
	(Dwarf Cinquefoil)		R^2^ = 0.00	2012: 90–147	2012: 105
Massachusetts	*Prunus pensylvanica*	22	y = −2.95x+147	2010: 100–128	2010: 105
	(Pin Cherry)		R^2^ = 0.29***	2012: 101–129	2012: 109
Massachusetts	*Prunus serotina* *	22	y = −2.08x+149	2010: 91–161	2010: 129
	(Black Cherry)		R^2^ = 0.04	2012: 92–161	2012: 131
Massachusetts	*Prunus virginiana*	24	y = −4.06x+165	2010: 92–138	2010: 122
	(Chokecherry)		R^2^ = 0.41***	2012: 94–139	2012: 123
Massachusetts	*Rhododendron canadense*	26	y = −4.27x+160	2010: 104–122	2010: 124
	(Rhodora)		R^2^ = 0.72***	2012: 106–123	2012: 123
Massachusetts	*Saxifraga virginiensis*	26	y = 0.81x+103	2010: 92–131	2010: 102
	(Early Saxifrage)		R^2^ = 0.02	2012: 93–130	2012: 92
Massachusetts	*Senecio aureus*	26	y = −2.36x+156	2010: 111–150	2010: 129
	(Golden Ragwort)		R^2^ = 0.13**	2012: 112–150	2012: 123
Massachusetts	*Silene caroliniana*	26	y = −3.85x+169	2010: 115–137	2010: 129
	(Wild Pink)		R^2^ = 0.58***	2012: 117–138	2012: 128
Massachusetts	*Smilax rotundifolia*	21	y = −4.12x+183	2010: 109–166	2010: 124
	(Common Greenbriar)		R^2^ = 0.19***	2012: 111–166	2012: 128
Massachusetts	*Trientalis borealis*	25	y = −4.43x+165	2010: 103–130	2010: 115
	(Starflower)		R^2^ = 0.53***	2012: 105–131	2012: 118
Massachusetts	*Trillium cernuum*	25	y = −2.84x+155	2010: 107–142	2010: 122
	(Nodding Trillium)		R^2^ = 0.21**	2012: 108–142	2012: 131
Massachusetts	*Vaccinium angustifolium*	26	y = −4.41x+152	2010: 88–118	2010: 105
	(Lowbush Blueberry)		R^2^ = 0.63***	2012: 90–119	2012: 98
Massachusetts	*Vaccinium corymbosum*	26	y = −6.55x+170	2010: 83–113	2010: 97
	(Highbush Blueberry)		R^2^ = 0.66***	2012: 85–115	2012: 92
Massachusetts	*Viola cucullata*	25	y = −3.28x+140	2010: 86–122	2010: 102
	(Marsh Blue Violet)		R^2^ = 0.27***	2012: 88–122	2012: 98
Massachusetts	*Viola fimbriatula*	23	y = −2.91x+142	2010: 86–134	2010: 102
	(Arrowleaf Violet)		R^2^ = 0.13**	2012: 88–135	2012: 105
Massachusetts	*Viola lanceolata*	24	y = −3.17x+150	2010: 100–130	2010: 120
	(Lance-leaved Violet)		R^2^ = 0.33***	2012: 101–130	2012: 115
Massachusetts	*Viola pedata* *	23	y = 2.22x+110	2010: 113–157	2010: 124
	(Birdfoot Violet)		R^2^ = 0.10	2012: 113–155	2012: 123
Wisconsin	*Amelanchier arborea* *	47	y = −4.85x+155	84–108	84
	(Serviceberry)		R^2^ = 0.63***		
Wisconsin	*Anemone canadensis*	47	y = −4.05x+179	116–144	111
	(Meadow Anemone)		R^2^ = 0.46***		
Wisconsin	*Anemone patens*	47	y = −3.31x+127	70–103	75
	(Pasque Flower)		R^2^ = 0.30***		
Wisconsin	*Anemone quinquefolia* *	47	y = −4.31x+149	84–109	87
	(Wood Anemone)		R^2^ = 0.55***		
Wisconsin	*Aquilegia canadensis* *	47	y = −3.98x+162	102–125	117
	(Wild Columbine)		R^2^ = 0.55***		
Wisconsin	*Arabis lyrata*	47	y = −3.84x+140	80–105	80
	(Sand Cress)		R^2^ = 0.49***		
Wisconsin	*Caltha palustris* *	47	y = −2.64x+134	91–112	100
	(Marsh Marigold)		R^2^ = 0.41***		
Wisconsin	*Dicentra cucullaria*	47	y = −4.46x+140	72–100	76
	(Dutchman's Breeches)		R^2^ = 0.52***		
Wisconsin	*Dodecatheon meadia*	47	y = −3.73x+163	110–126	122
	(Shooting Star)		R^2^ = 0.70***		
Wisconsin	*Fragaria virginiana* *	47	y = −3.93x+154	90–123	102
	(Wild Strawberry)		R^2^ = 0.38***		
Wisconsin	*Geranium maculatum* *	47	y = −4.52x+165	98–122	111
	(Wild Geranium)		R^2^ = 0.59***		
Wisconsin	*Hepatica nobilis*	47	y = −4.18x+132	64–98	75
	(Sharp-lobed Hepatica)		R^2^ = 0.40***		
Wisconsin	*Lithospermum canescens*	47	y = −4.13x+161	96–126	105
	(Hoary Puccoon)		R^2^ = 0.44***		
Wisconsin	*Oxalis stricta*	47	y = −4.23x+167	104–126	118
	(Wood Sorrel)		R^2^ = 0.62***		
Wisconsin	*Phlox divaricata*	47	y = −5.38x+167	86–116	94
	(Woodland Phlox)		R^2^ = 0.57***		
Wisconsin	*Phlox pilosa*	47	y = −3.66x+166	106–136	128
	(Prairie Phlox)		R^2^ = 0.38***		
Wisconsin	*Prunus serotina* *	47	y = −3.74x+167	109–134	126
	(Black Cherry)		R^2^ = 0.49***		
Wisconsin	*Rubus allegheniensis*	47	y = −3.01x+169	124–140	129
	(Common Blackberry)		R^2^ = 0.63***		
Wisconsin	*Sanguinaria canadensis*	47	y = −3.55x+129	71–100	76
	(Bloodroot)		R^2^ = 0.40***		
Wisconsin	*Sisyrinchium campestre*	47	y = −3.83x+165	108–129	118
	(Blue-eyed Grass)		R^2^ = 0.58***		
Wisconsin	*Tradescantia ohiensis*	47	y = −3.27x+174	119–149	131
	(Spiderwort)		R^2^ = 0.34***		
Wisconsin	*Trillium grandiflorum*	47	y = −2.58x+142	93–128	99
	(Large-flowered Trillium)		R^2^ = 0.19***		
Wisconsin	*Viola pedata* *	47	y = −5.11x+164	91–112	101
	(Birdfoot Violet)		R^2^ = 0.70***		

The samples size is the number of years used for each regression analysis. Species names follow the United States Department of Agriculture Plants Database. Species common to both locations are indicated with an asterisk after the species name. Asterisks that follow R^2^ values represent significance at the following levels:

* = p<0.05,

** = p<0.01,

*** = p<0.001.

**Figure 2:**
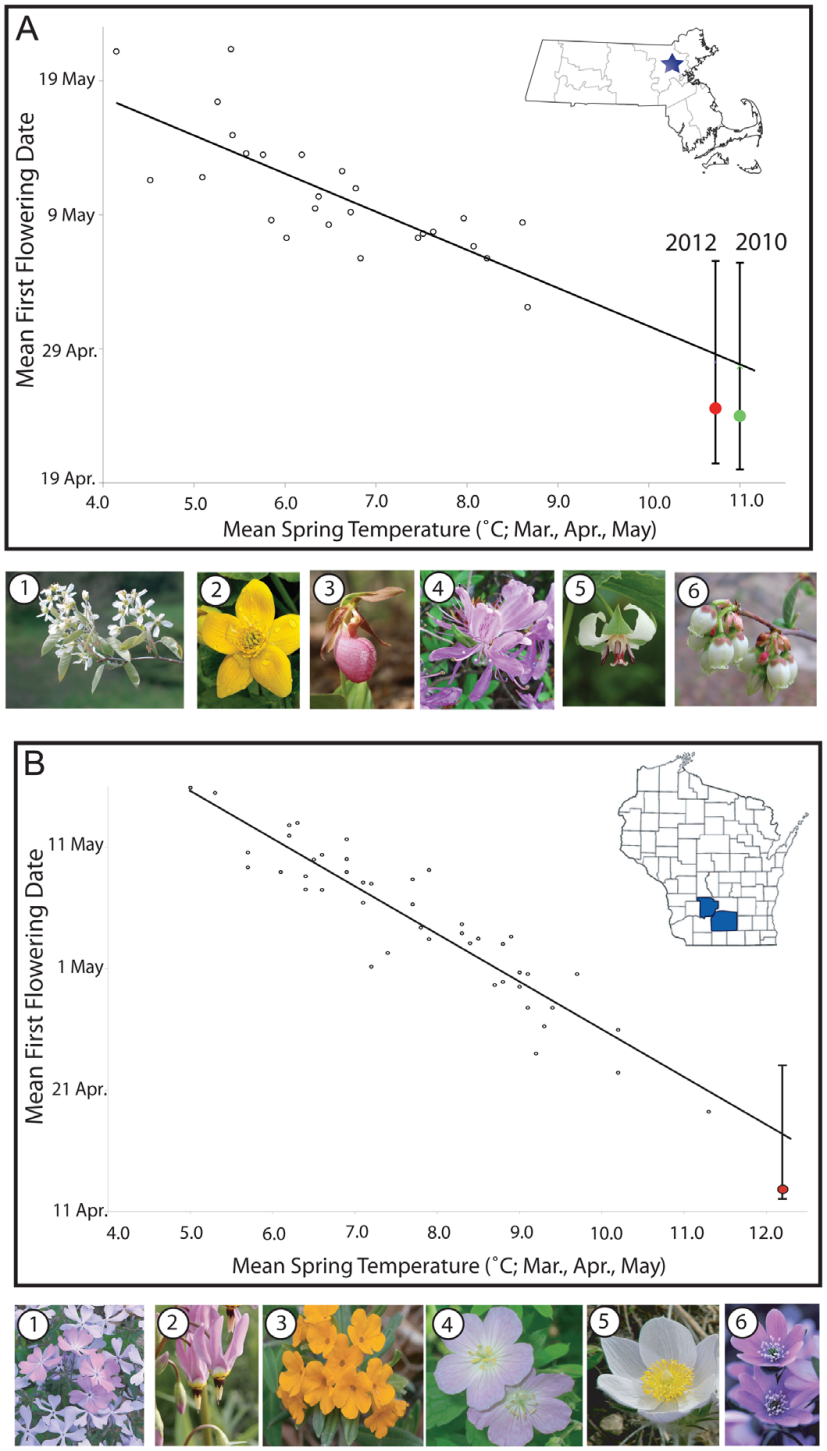
The relationships between mean first flowering dates and mean spring (March, April and May) temperatures. Each dot represents the mean first flowering date of all sampled species for a given year in (a) Massachusetts and (b) Wisconsin. Black regression lines, and 95% prediction intervals, were estimated from pre-2010 data (Massachusetts) and pre-2012 data (Wisconsin). 2012 observed values are shown in solid red, and 2010 (Massachusetts only) in green. The 95% prediction intervals for 2010 and 2012 mean first flowering dates are indicated with vertical lines. Photographs illustrate representative species at each location. Unless specified otherwise, photographs are made available under an Attribution-Share Alike 2.0 License with date and photographer as listed. Massachusetts species: 1) serviceberry (*Amelanchier canadensis*), © 2011 personal collection of R. Primack, 2) marsh marigold (*Caltha palustris*), © 2009 walker_bc, 3) pink lady slipper (*Cypripedium acaule*), © 2012 Graham Hunt, 4) rhodora (*Rhododendron canadense*), © 2012 Andrew Block, 5) nodding trillium (*Trillium cernuum*), © 2008 Ed Post, and 6) highbush blueberry (*Vaccinium corymbosum*), © 2007 Anita363. Wisconsin species: 1) woodland phlox (*Phlox divaricata*), © 2009 Diane DiOhio, 2) shooting star (*Dodecatheon meadia*), © 2006 Frank Mayfield, 3) hoary puccoon (*Lithospermum canescens*), © 2006 cotinis, 4) wild geranium (*Geranium maculatum*), © 2009 aposematic herpetologist, 5) pasque flower (*Anemone patens*) © 2007 Malcom Manners, and 6) sharplobe hepatica (*Hepatica nobilis*) © 2009 Alan J. Hahn.

Numerous species in Massachusetts have shown remarkable shifts in flowering times in recent years [27], [30]. In 2010, 13 of the 32 species we analyzed had their earliest flowering date on record. In 2012, a different 14 species had their earliest recorded flowering date. Thoreau, for example, observed highbush blueberry (*Vaccinium corymbosum*) flowering in mid-May (11–21 May). In 2012 this species flowered on 1 April, six weeks earlier than observed by Thoreau. Based on our linear regression analysis of these historical phenology and temperature data, plant species flower on average 3.2 days earlier for each 1°C rise in mean spring temperatures (Figure 2a, p<0.001, R^2^ = 0.75). Twenty-seven of these 32 species exhibit significantly (p<0.05) earlier flowering times with spring temperatures (Table 1). Our results are robust to phylogenetic relationships: when phylogeny was incorporated into a generalized least squares analysis of phenological response to spring temperature, the results remained highly significant (P<0.01).

In south-central Wisconsin, 23 phylogenetically diverse spring-flowering native plant species have been monitored in each of the following 47 years: 1935–1945 and 1977–2012 (Fig. 1b; Table 1, and phylogenetic relationships in Figures S1a and S1b). During this time, Wisconsin's spring temperatures have warmed dramatically as a result of climate change [31]. During 1935–1945, when mean spring temperature was 7.5°C, the mean flowering date was 7 May. During the most recent 11-year period (2002–2012), when mean spring temperature was 9.3°C, the mean flowering date advanced by 7 days to 1 May. The mean spring temperature in 2012 was 12.2°C, the warmest on record and substantially warmer than the previous high of 11.3°C in 1977. In 2012, mean flowering was 13 April, the earliest date ever recorded, and over 3 weeks earlier (i.e., 24 days) than mean flowering in Leopold's years.

Most species in Wisconsin showed dramatic shifts in their flowering dates during this time. In 2012, 19 of the 23 species equaled or surpassed their previous earliest flowering dates. This response has been especially strong for several species. For example, Leopold recorded the first flower of woodland phlox (*Phlox divaricata*) between 28 April and 27 May; in 2012 it flowered on 4 April. Likewise, he recorded serviceberry (*Amelanchier arborea*) flowering between 10 April and 9 May; in 2012 it flowered on 25 March. Based on our analyses of these cumulative phenology and temperature data, plants in south-central Wisconsin flower on average 4.1 days earlier for each 1°C rise in mean spring temperature (Figure 2b, p<0.001, R^2^ = 0.88). All 23 species exhibit significantly (p<0.05) earlier flowering times with warming spring temperatures (Table 1). As in Massachusetts, our results were robust to phylogenetic relationships (P<0.05).

Given the significant relationship between mean spring temperatures and mean first flowering dates, the recent record-breaking warm springs of 2010 and 2012 in Massachusetts and 2012 in Wisconsin provide an opportunity to test whether historical relationships predict mean flowering dates during these exceptionally warm years. Based on regression analyses of pre-2010 data (Massachusetts) and pre-2012 data (Wisconsin), the mean observed first flowering dates for the focal species during 2010 and 2012 fell within the 95% prediction intervals at each location (Figure 2) [32]. These prediction intervals [30] are estimates of the range of dates within which 2010 and 2012 observations of mean first flowering date are expected to fall, within a 95% probability. Results for individual species were also similar (Table 1). For the 32 species in Massachusetts, all but two flowered within the prediction interval for 2010. Marsh marigold [*Caltha palustris*] flowered earlier, and rhodora [*Rhododendron canadense*] flowered later than predicted. In 2012, only early saxifrage [*Saxifraga virginiensis*] flowered earlier than predicted. For Wisconsin, 22 of the 23 species had flowering times in 2012 that were within the 95% prediction intervals. Meadow anemone (*Anemone canadensis*) was the lone outlier, flowering five days earlier than the predicted interval. These results indicate that spring-flowering plants at both locations, whether analyzed as single species or averaged across all species, largely responded to record-breaking warm temperatures as predicted by their historical responses to warming spring temperatures.

These results collectively demonstrate that despite record-breaking warm temperatures in the eastern United States, plants have continued to flower earlier in the face of recent dramatic climate change. While other studies have examined long-term observations with comparable rates of phenological advancement [2], [3], [33], [34], to our knowledge ours is the first to demonstrate the predictive power of such data under unprecedented warm temperatures. In contrast to our results, there is increasing discussion in the literature [6]–[8] that flowering, leaf out, and growth could be delayed for temperate plants that have not experienced lengthened spring photoperiods or extended cool temperatures that satisfy their winter chilling requirements. A delay in phenology caused by insufficient chilling is most likely to be observed first in warm temperate latitudes where winter temperatures are barely adequate for fulfilling chilling requirements for some species [8], [35]. Another scenario is highlighted in a recent study [7] suggesting that individual species thought to be unresponsive to spring temperature were actually responding to both an unsatisfied chilling requirement and warmer spring temperatures resulting in no net change in flowering phenology. Based on our results, there is no indication that the 47 spring flowering plants we studied are delayed in their flowering by insufficient photoperiod or winter chilling requirements. These plants continue to flower earlier apparently in direct response to increasingly warmer mean spring temperatures (R^2^ values = 0.75–0.88). Other climatic factors such as late winter temperatures or spring minimum temperatures may exert some effects, but we did not detect them here. This strongly suggests that most of these plants have not yet reached a physiological threshold.

By extension, because flowering and leaf-out times are highly correlated for many species [36], [37], we hypothesize that yet earlier flowering times, and potentially leaf out times, will continue to be observed in the face of predicted climate change. In contrast to a number of phenological studies showing nonlinear relationships between phenology and temperature, due largely to unmet chilling and photoperiod requirements, our findings demonstrate the relationship to be linear and to explain most of the variation in flowering. It is possible of course, that these observations are within a fairly linear portion of a relationship that will prove to be nonlinear with future climate change [38] [39]. As temperatures continue to rise in the northeastern United States this linearity of the relationship of flowering time to temperature will be tested. Importantly, on-going ecological monitoring initiated by Thoreau and Leopold will help to clarify the complexities of this system under future change, and to illuminate plant phenological responses in experimental warming plots and under greenhouse conditions.

## Materials and Methods

### Phenological and climate data

Observations of first flowering dates of species in Concord, Massachusetts, USA (42°27′37″N, 71°20′58″W) were made by Thoreau during the years 1852–1858, Hosmer for 1878 and 1888–1902, Primack, Miller-Rushing and their associates for 2003–2006, and Primack and his associates for 2008–2012 [24]. Thirty-two spring-flowering native species from a variety of habitats were chosen from a list of over 200 species because of the criterion of being observed in nearly all years. At the Massachusetts site, *Amelanchier arborea* and *A. canadensis* cannot be readily distinguished and flower at the same time; for convenience these combined observations are listed under the name *A. arborea*. This dataset includes all species that met these criteria, while non-native species, species with few observations and summer-flowering species were not included in this analysis (Table 1). These data are available on the Primack Lab website (people.bu.edu/primack). Phenological observations were made on both public and private lands; permission was obtained for private land when necessary. No permission was needed for public lands. No destructive tissue sampling was conducted. Temperature data are from Blue Hill Meteorological Observatory in East Milton, Massachusetts and are available through NOAA National Climatic Data Center (http://www.ncdc.noaa.gov/oa/ncdc.html) [40]. Blue Hill Meteorological Observatory is located 33 km southeast of Concord, MA and temperatures between the two nearby locations are highly correlated [27].

Leopold, his family members, and his students collected phenological data from 1935–1945 at locations in Sauk and Dane Counties, Wisconsin, USA, primarily near the Leopold “Shack” (43°33′46″N, 89°39′33″W) and in the University of Wisconsin Arboretum (43°02′48″N, 89°24′58″W). NLB, SAT, and the staff of the Aldo Leopold Foundation collected phenological data from 1977–2012 at locations in Sauk and Dane Counties primarily near the Leopold shack and in Dunlap Hollow (43°12′12″N, 89°45′06″W). Twenty-three spring-flowering native species were chosen from a list of 176, for which observations of first flowering had been made in every year. These data are available by contacting SAT. Permits and approvals were not necessary for the private lands where observations were made in Wisconsin, or for public property of the University of Wisconsin Arboretum. None of the Wisconsin species observed in this study have protective status, and no destructive sampling was conducted. Mean spring temperatures for the south-central Wisconsin climatic region, which includes our study sites, were obtained from the Wisconsin State Climatology Office (http://www.aos.wisc.edu/~sco/clim-history/division/data/temp/WI-08-TEMP.xls).

### Statistical analysis and phylogenetic methods

Mean annual temperatures for those months that best predict spring flowering times were used in this analysis (i.e., March, April and May). April and May are the predominant flowering months for these species, and the inclusion of March temperatures strengthened the model. Mean temperatures for this time period provided the strongest model, owing to the fact that plants are accumulating heat and beginning spring growth. While certain studies have shown that the inclusion of winter months improves the relationship between flowering and temperature [41], we did not find that to be the case with this data set. For example, the model of flowering in Concord using only mean monthly April and May temperatures provided a strong model (R^2^ = 0.70), yet including May temperatures explained an even larger amount of variation (R^2^ = 0.75). Adding mean February temperature weakened this relationship (R^2^ = 0.71); using mean monthly temperatures from January through May weakened this relationship further (R^2^ = 0.64).

We performed all analyses in R 2.15.1 [42]. We calculated linear regressions (mean first flowering date for all species over time as well as mean first flowering date for each species versus mean spring temperature) for all years at both study sites, respectively.

We used mean spring temperature rather than another index of spring (e.g., growing degree days) due to the ease of calculating, displaying, and explaining this variable. Also, this simple measure of spring temperature explained most of the variation in flowering times. To test the linearity of the relationship between temperature and flowering time, we analyzed the residuals of this relationship and found them to be well scattered in a random pattern. This indicates that the relationship is consistent and that flowering is not earlier or later over time other than expected relative to temperature.

We also performed statistical comparisons to account for phylogenetic non-independence. Two highly resolved dated phylogenies were produced for each of the two sites to accomplish this goal (see Text S1 and Figures S1a and S1b). We did not conduct a multiple model regression test, but have previously shown in such an analysis using the Concord data that phenological response and abundance change is most strongly tied to changes in temperature [26]. All phylogenies and data matrices are available on TreeBase. Traits at both locations did not exhibit phylogenetic conservation as determined by Blomberg's K in the *picante* package version 1.4–2 (K<1.00) [43]. This indicates that the patterns we observed are not caused by groups of related species possessing similar traits. Trait correlations as above were tested using a phylogenetic general linear model as implemented using the pgls function in the *caper* package version 0.5. This model includes a variance-covariance structure based on evolutionary distance to control for phylogenetic non-independence in the data [44].

To determine prediction intervals that excluded recent record-breaking warm years, we recalculated linear regressions using only pre-2010 observations (for Massachusetts) and pre-2012 observations (for Wisconsin). Then, we calculated the 95% prediction intervals for mean first flowering dates for all species and flowering dates for each species for Massachusetts (separately for 2010 and 2012, using only pre-2010 observations) and Wisconsin (for 2012), based on the observed mean spring temperatures for those record-breaking warm years [32]. We then compared the observed mean first flowering dates for all species and flowering dates for each species in 2010 and 2012 (in Massachusetts) and 2012 (in Wisconsin) with those predictions.

Eight species were common to both sites and allow us to compare their responses to temperature (Table 1). An analysis of covariance (ANCOVA) was used to determine if location influenced how first flowering dates varied over time and in response to temperature. We then tested whether the regression lines of the relationship between year and first flowering date were the same between the two locations. This was repeated for the relationship between temperature and first flowering date for these common species. Mean flowering times varied over years in a similar way at both locations (ANCOVA F _1, 75_ = 2.6, p = 0.427). However, their responses to temperature differed between locations (ANCOVA F _1, 75_ = 69.1, p<0.001). The contrasting responses to temperature may be related to multiple factors, including local adaptation to temperature and other related climate variables, or sampling issues including changes in species' abundance at each location [45], [46]. Future observational studies and transplant experiments of these species will help us to better understand these differences.

## Supporting Information

Figure S1 **S1a.** Phylogeny of Massachusetts spring-flowering plant species used in the analyses. **S1b.** Phylogeny of Wisconsin spring-flowering plant species used in the analyses.
(TIF)

Text S1 **Phylogenetic analysis description and methods.**
(DOCX)
