# Supplementary material for: Record-Breaking Early Flowering in the Eastern United States
Source: PLoS One. 2013 Jan 16;8(1):e53788. doi: 10.1371/journal.pone.0053788 (PMC3547064; doi:10.1371/journal.pone.0053788)
Supplement: Text S1 — Phylogenetic analysis description and methods. (DOCX) [file pone.0053788.s002.docx]

**Supporting Information: Text S1**

Phylogeny for the Massachusetts and Wisconsin spring floras

We first assembled two phylogenies for all of the species at each of our two sites using Phylomatic [1], which assumes the APG III angiosperm phylogeny backbone [2] (See Supporting Information Figures 1a and b). We additionally resolved a small number of unresolved relationships in these trees using DNA sequence data from GenBank. To do this we used a mega phylogeny inferred for a study on the relationship between phylogenetic distance on and plant species’ invasiveness (Schaefer et al., *in prep*). GenBank nucleotide data for 17 DNA regions were downloaded and data matrices for the entire flora of the continental United States were assembled and aligned using the PHLAWD computer package [3]. These DNA matrices were then analyzed using Maximum Likelihood as implemented in RAxML v7.2.8 [4]. Here, we greatly reduced this mega phylogeny to produce phylofloras for each of our two sites using the name.check function in the *geiger* package version 1.3-1 and the drop.tip function in the *ape* package version 3.0-5 [5]. These phylogenies were then used to resolve the remaining nodes in the Phylomatic tree described above, most of which were within a small number of genera. Finally, divergence times were then estimated for each of these phylogenies using dates from Wikstrom et al. [6] as implemented using the bladj algorithm of Phylocom [7]. The tree files for each location are available on the Primack Lab website.

REFERENCES

1 Webb CO, Donoghue MJ (2005) Phylomatic: tree assembly for applied phylogenetics. Molecular Ecology Notes 5(1): 181-183.

2 Bremer B, Bremer K, Chase M (2009) An update of the Angiosperm Phylogeny Group classification for the orders and families of flowering plants: APG III. Botanical Journal of the Linneaen Society 161(2): 105-121.

3 Smith SA, Beaulieu J, Donoghue MJ (2009) Mega-phylogeny approach for comparative biology: an alternative to supertree and supermatrix approaches. BMC Evolutionary Biology 9: 37.

4 Stamatakis A, Hoover P, Rougemont J (2008) A fast bootstrapping alogorithm for the RAxML web-servers. Systematic Biology 57(5): 758-771.

5 Paradis E, Claude J, Strimmer K (2004) APE: Analyses of Phylogenetics and Evolution in R language. Bioinformatics 20(2): 289-290.

6 Wikstrom N, Savolainen V, Chase MW (2001) Evolution of angiosperms: calibrating the family tree. Proceedings of the Royal Society B-Biological Sciences 268: 2211-2220.

7 Webb CO, Ackerly DD, Kembel SW (2008) Phylocom: software for the analysis of phylogenetic community structure and trait evolution. Bioinformatics 24: 2098-2100.

**Supporting Information Figure S1a.** Phylogeny of Massachusetts spring-flowering plant species used in the analyses.

**Supporting Information Figure S1b.** Phylogeny of Wisconsin spring-flowering plant species used in the analyses.
